# Case Series: Myelin Oligodendrocyte Glycoprotein-Immunoglobulin G-Related Disease Spectrum

**DOI:** 10.3389/fneur.2020.00089

**Published:** 2020-02-26

**Authors:** Foziah Alshamrani, Hind Alnajashi, Eslam Shosha, Courtney Casserly, Sarah A. Morrow

**Affiliations:** ^1^Department of Neurology, King Fahad University Hospital, Imam Abdulrahman Bin Faisal University, Dammam, Saudi Arabia; ^2^Department of Neurology, King Abdulaziz University, Jeddah, Saudi Arabia; ^3^Department of Clinical Neurological Sciences, University of Western Ontario, London, ON, Canada

**Keywords:** myelin oligodendrocyte glycoprotein, optic neuritis, neuromyelitis optica spectrum disorder, multiple sclerosis, transverse myelitis

## Abstract

**Introduction:** Myelin oligodendrocyte glycoprotein-immunoglobulin G (MOG-IgG)-related disease was initially described as a subtype of neuromyelitis optica spectrum disorder (NMOSD) with antibodies against MOG. However, it has recently been described as a separate disease entity with clinical and radiological features that overlap those of multiple sclerosis (MS) and NMOSD; the clinical features of this disease phenotype remain undetermined. We herein report the clinical presentation of nine MOG-IgG-positive patients, not all of whom fulfill the NMOSD criteria, in order to highlight the features and challenges of this condition.

**Method:** We retrospectively reviewed the records of the London (Ontario) MS clinic to identify patients diagnosed with positive MOG antibodies based on the 2015 NMOSD consensus criteria.

**Result:** Nine patients were identified, all Caucasian. Seven (78%) were female, and the median age of onset was 41 years (range, 28–69 years); the median Expanded Disability Status Scale score at onset was 3.0 (range, 2.0–4.0). A monophasic course was noted in two (22.2%) patients, while the median number of relapse events was 3 (range 2–5) in 77.8% of the patients. Optic neuritis and transverse myelitis contributed equally as initial manifestations in three individuals (33%), while brainstem relapse was reported in two individuals (22%). The brain magnetic resonance imaging findings were compatible with McDonald's 2010 dissemination in space criteria in three cases (33%). Short myelitis and an (H)-sign were each documented in one patient.

**Conclusion:** The phenotypes of MOG Ab-positive cases exhibited overlapping features with MS and NMOSD. This finding highlights the importance of screening for anti-MOG in individuals with demyelinating symptoms, in consideration of the possibility of false-positive MOG Ab results.

## Introduction

Myelin oligodendrocyte glycoprotein (MOG) is a component of central nervous system (CNS) myelin. Antibodies against MOG have recently been recognized in a clinical syndrome that is likely a CNS demyelinating disorder separate from multiple sclerosis (MS), acute demyelinating encephalomyelitis (ADEM), and neuromyelitis optica spectrum disorder (NMOSD). Although MOG antibodies have been mentioned in the literature for the last 30 years, their role in demyelinating disease has not been fully elucidated and, to date, remains controversial (1, 2). In experimental allergic encephalomyelitis mouse models, MOG is the only CNS myelin autoantigen to cause both an encephalitogenic T cell-mediated inflammatory response and demyelination (3, 4). The significance of this is unclear, and the prevalence of MOG antibodies in MS remains undetermined.

MOG antibodies have recently been linked to seronegative cases of NMOSD. Recent cohort studies have demonstrated that 15–35% of seronegative NMOSD patients will test positive for MOG antibodies (5). The presence of MOG antibodies is not only described in seronegative cases of NMOSD (6); indeed, MOG antibody-positive cases have also been identified within a wider spectrum of demyelinating disorders. Recurrent optic neuritis, myelitis, brainstem encephalitis, and ADEM-like presentation such as encephalomyelitis have all been described in MOG-immunoglobulin (IgG)-positive patients (7–9). However, the clinical features of this disease phenotype remain undetermined. We herein report the clinical presentation of a case series of MOG-IgG-positive patients, not all of whom fulfill the NMOSD criteria, in order to highlight the features and challenges of this condition.

## Case Description

This study was approved by the University of Western Ontario's (Western) Health Science Research Ethics Board and written informed consent was obtained from all patients.

All individuals who tested positive for anti-MOG at the London (Ontario) MS clinic were retrospectively reviewed. Data were obtained for age at onset, sex, first clinical presentation, number of relapses, disease course, and duration. The neurological examination data included the Expanded Disability Status Scale (EDSS) score at the initial and final follow-up and brain and spine magnetic resonance imaging (MRI). In addition, data on serological testing and cerebrospinal fluid (CSF) analysis including oligoclonal bands (OCB) were collected if available. Data on current and disease-modifying therapies (DMTs) were also included. Nine MOG-IgG-positive cases were identified (Table 1).

**Table 1:** Demographic, clinical, and radiological characteristics of patients.

**Case**	**Age**	**Sex**	**Initial symptoms**	**Relapse #**	**Initial EDSS**	**Final visit EDSS**	**Brain MRI**	**Spine MRI**	**CSF OCB**	**Long-term treatment**
A	52	M	Brainstem (vertigo)	3	2	1	Multiple periventricular and deep white matter lesions	N/A^*^	3 OCB	AZT^**^
B	29	F	Brainstem (diplopia and ataxia)	2	2	2	Left optic nerve enhancement	Normal	Negative	Mycophenolic acid
C	31	F	Short myelitis	2	3	2	Multiple supratentorial and infratentorial lesions	Multiple cervical and thoracic segment (2-3 vertebral lengths)	N/A	Was on glatiramer acetate, discontinued and received no further treatment
D	28	M	ON^***^	3	4	2	Right optic nerve hyperintensity up to the chiasma and enhancement	N/A	Negative	Mycophenolic acid
E	43	F	ON	4	4	2	Right optic nerve hyperintensity, no contrast enhancement	Normal	Negative	No treatment
F	58	F	Bladder and ataxia	1	3.5	2	Few subcortical hyperintensities	Normal	Negative	AZT
G	69	F	ON	4	2.5	3	Juxtacortical, periventricular, and deep white matter more pronounced in both occipital lobes	Normal	Negative	No treatment
H	34	F	Transverse myelitis	1	2.5	1	Normal	Longitudinally extensive hyperintensity in the thoracic spinal cord	Negative	No treatment
I	35	F	Longitudinal transverse myelitis	1	3.5	2	Normal	Longitudinally extensive hyperintensity in the cervical spinal cord	Negative	No treatment

* N/A, not available;

** AZT, azathioprine;

*** *ON, optic neuritis; OCB, oligoclonal bands; EDSS, Expanded Disability Status Scale; MRI, magnetic resonance imaging; CSF, cerebrospinal fluid*.

### Case A

**Figure 1:**
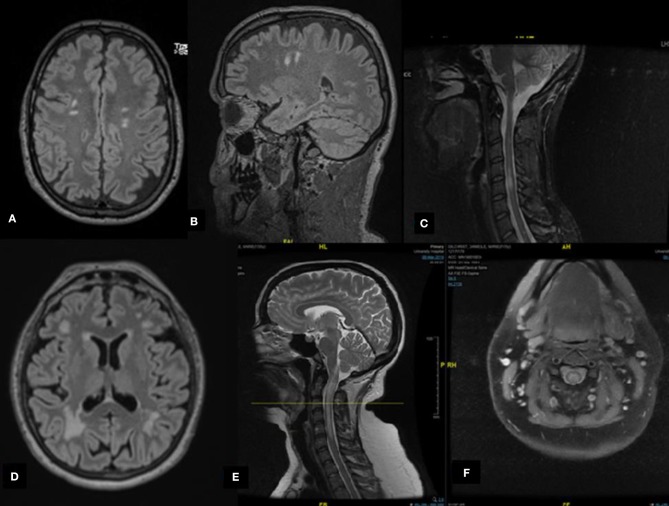
Radiological features of selected cases. **(A,B)** Case A: Axial and sagittal magnetic resonance imaging (MRI) fluid attenuated inversion recovery (FLAIR) revealing multiple subcortical hyperintense lesions. **(C)** Case C: Sagittal MRI short-TI inversion recovery of the cervical spine demonstrates cervical hyperintensities. **(D)** Case G: Axial MRI FLAIR revealing multiple hyperintense lesions involving subcortical and periventricular areas, predominantly in the occipital regions. **(E,F)** Case I: Cervical spine MRI T2 reveals a hyperintense lesion extending from the posterior medulla (area postrema) to the area between C5 and C6 of the spinal cord forming an H sign.

A 52-year-old male patient was referred due to suspected MS. In 2008, he presented with an episode of vertigo and gait instability, which resolved over a period of 2 months following corticosteroid and plasmapheresis treatment. He remained quiescent until 2017, when he presented with right facio-brachial weakness for 3 weeks. The EDSS score was 1.0 at the final follow-up at the clinic in 2019; brain MRI confirmed McDonald's 2010 dissemination in space (DIS) criteria (Figure 1). CSF analysis revealed one distinct band and two faint bands with a normal IgG index. One year later, the patient experienced sensory spinal cord relapse and was started on azathioprine. No spinal cord lesions were identified on 1.5-T MRI at our center.

### Case B

A 29-year-old female patient presented in 2008 with double vision, ataxia, nausea, and vomiting. She was presumptively diagnosed with Miller Fisher syndrome or possibly thiamine deficiency; she was treated with intravenous immunoglobulin and thiamine, and her condition returned to normal within 3 months. All serology at that time was unremarkable. CSF analysis was unremarkable without albumin-cytological dissociation during hospitalization.

In 2017, she developed decreased vision and color perception in her left eye; her visual acuity (VA) was 20/20 in the right eye and 20/50 in the left, with left relative afferent pupillary defect (RAPD). She scored 12/16 on the Ishihara color plate on the left eye, and the EDSS score was 2.0. She partially responded to 5 days of intravenous (IV) methylprednisolone treatment. Her brain MRI revealed an enhanced lesion at the anterior aspect of the left optic nerve sheath with unremarkable brain and spine findings. Her CSF examination was negative for OCB, and she had a normal IgG index. She was maintained on mycophenolic acid with no further relapse or new MRI brain lesions to date. At her final follow-up in 2019, her EDSS score was 2.0.

### Case C

A 31-year-old female patient was referred to our clinic with a diagnosis of MS following a diagnosis of transverse myelitis in 2014. Her spine MRI revealed short and long myelitis in the cervical and thoracic spinal cord (Figure 1). She was treated with IV methylprednisolone for 5 days with no significant improvement. She was subsequently treated with a course of plasma exchange. She exhibited some improvement, but residual mild right-sided weakness remained. The EDSS score improved from 3.0 to 2.0 post-treatment. The patient was maintained on glatiramer acetate. One year later, she presented with optic neuritis. Brain MRI confirmed the DIS criteria. She discontinued glatiramer acetate in 2019 post-MOG testing and preferred not to start any further DMTs. Her disease remained clinically and radiologically inactive. The EDSS score remained steady at 2.0 at her final follow-up in 2019.

### Case D

A 19-year-old male patient presented in 2008 with right optic neuritis with residual peripheral visual field defect. In 2017, he presented with another episode of severe right optic neuritis; his VA in that eye was finger counting only. The EDSS score at this time was 4.0. Fundoscopic examination revealed bilateral optic pallor. There was no RAPD. He was treated with a 5-day course of IV methylprednisolone with good recovery; his uncorrected VA was 20/20 on the left and 20/40 on the right. His brain MRI revealed increased signal in his right optic nerve reaching the optic chiasm with mild gadolinium enhancement, with no brain or spinal cord lesions. His CSF examination was negative for OCB, and he had a normal IgG index. He was maintained on mycophenolic acid with no further relapses or imaging activity. At the final follow-up in 2018, the EDSS score was 2.0.

### Case E

A 43-year-old female patient presented with right optic neuritis since September 2014, with recurrent attacks in July 2015, December 2015, and June 2016. Her VA during each attack was 20/100 in the right eye and 20/20 in the left eye. She received 3 days of IV corticosteroids followed by oral prednisone treatment for her first episode in September 2014, and 1,250 mg of prednisone for 3 days followed by a tapered dose for the 2015 and 2016 relapses. After treatment, her corrected VA was 20/30-2 in the right eye and 20/20-2 in the left eye, with right RAPD. The brain and spine MRI examinations were unremarkable. Her CSF examination was negative for OCB with a normal IgG index. After treatment, her EDSS score improved from 4.0 to 2.0 with no further disease activity and normal brain MRI findings until 2018, despite no DMT upon patient preference.

### Case F

A 58-year-old female patient presented in February 2018 with ataxic gait along with bladder and bowel urgency and incontinence. She was initially evaluated by a urologist and received an undetermined diagnosis. Her neurological assessment revealed severe gait and truncal ataxia, and an EDSS score of 3.5. Her brain MRI indicated two foci of increased T2 signal in the subcortical area, and these lesions were non-specific. No lesions were identified in the spinal cord. Her CSF examination was negative for OCB, and she had a normal IgG index. She did not receive any first-line therapy and was maintained on azathioprine with disease stability but a subtle increase in the size of the T2 lesions. The EDSS score at the final follow-up in March 2019 had worsened from 3.5 to 2.0.

### Case G

A 69-year-old female patient who experienced two separate relapses of optic neuritis, one in each eye, with partial spontaneous recovery, re-presented in January 2018 with right arm weakness. She received 1,250 mg of prednisone for 3 days, but her right arm strength did not return to baseline. Her EDSS score was 2.5 according to visual and pyramidal findings. Her brain MRI was compatible with the DIS criteria. No cord lesions were identified (Figure 1). Her CSF examination was negative for OCB, and she had a normal IgG index. The patient declined to start DMT, despite worsening balance. At her final follow-up in 2018, her EDSS score was 3.0.

### Case H

A 34-year-old female patient presented in July 2018 with sensory symptoms, bladder frequency and urgency, along with L'hermitte phenomena. Her EDSS score was 2.5 according to the bladder symptoms and pyramidal findings. Her brain MRI was normal, but longitudinal extension was identified from T2 to T11. Her CSF examination was negative for OCB and she had a normal IgG index and slight increase in CSF proteins. The patient recovered with no treatment within 4 weeks, and her EDSS score improved to 1.0. Repeat MRI revealed subtle lesions at T2 in the spinal cord.

### Case I

A 35-year-old female patient presented with left-sided paresthesia and weakness, without bowel or bladder symptoms, that gradually progressed over the course of 2 weeks. There was no history of visual symptoms, nausea, vomiting, hiccups, or change in appetite. Her initial EDSS score was 3.5 according to the severe pyramidal and moderate sensory findings. Her spine MRI revealed an extensive lesion from the dorsal medulla to C6. On the axial T2 view, there was prominent gray matter involvement forming an H sign (Figure 1). Her brain MRI was unremarkable. CSF analysis revealed only lymphocytic pleocytosis, with a lack of high protein levels or intrathecal immunoglobulin synthesis. The patient's EDSS score improved to 2.0, with minimal pyramidal disability, 1 week after receiving 5 days of high-dose corticosteroids. Her serum anti-MOG was positive, and she was maintained on oral corticosteroids for 6 months.

## Discussion

Currently, there are no published diagnostic criteria for MOG-IgG-related disease. However, there are some recommendations based on expert consensus regarding appropriate testing for MOG antibody as well as some preliminary diagnostic criteria (10). Optic neuritis, either unilateral or bilateral, and myelitis are the most common presentations. Brainstem and supratentorial encephalomyelitis are also recognized presentations. Myelitis and optic neuritis occurred simultaneously in some of our cases (9). Generally, symptoms present as an acute demyelinating attack, while a progressive course seems to be extremely rare (9). Some unusual presentations have been reported in previous studies, for example, bilateral lower limb sensory symptoms with normal spine MRI findings (11) and symptoms suggestive of spinal involvement with no spinal lesions.

In an immune-mediated optic neuritis analysis (12), MOG-IgG1 was identified in 10% of those with single-episode isolated optic neuritis, 25% of those with recurrent isolated optic neuritis (RION), and 25% of those with chronic relapsing inflammatory optic neuropathy (CRION). These proportions were comparable to those in our cohort: 22% had RION, and 11% had CRION.

Area postrema syndrome/lesions have been identified as disease-defining symptoms for AQP4-positive NMOSD (13, 14); however, this syndrome/localization is not specific (14). In Case I, we observed extensive myelitis extending from the dorsal medulla to C6; however, the patient's history was unremarkable for intractable nausea, vomiting, or hiccups. While this is only a single case, it might suggest that the circumventricular body in the fourth ventricle is less impacted in MOG-IgG-related disease, while patients with anti-AQP4 and area postrema involvement may be more clinically unwell.

MOG-IgG-related disease more frequently presents with a relapsing course, although monophasic cases have been described. A monophasic course may be attributed to age and a short follow-up duration in previous studies. In a large cohort of 197 cases reported by Cobo-Calvo et al. (15), the cumulative risk of relapse after 2 and 5 years was 45 and 62%, respectively (15). MOG-IgG-related disease can also mimic MS in the form of recurrent relapsing attacks (9, 16). In our case series, 78% of the patients had a relapsing course; the median number of relapses was 3 (range, 2–5).

No patient has been reported to test positive for MOG-Ab and NMO-Ab simultaneously (4, 17). All of our patients were tested in-house using the Euroimmune commercial biochip immunofluorescence cell-based assay. Although there is a possibility of false-positive results in some of the cases, the risk is low as the Euroimmune cell-based assay has an 82.1% positive predictive value (18, 19).

Misdiagnosis of MOG-IgG-related disease as MS can be common. Among 16 MOG-IgG-related disease patients (20), 6 (37.5%) had MS-like syndromes (opticospinal disease with MS-like features, i.e., short lesions in the spinal cord, good recovery from optic neuritis, progression of disability between relapses), and 2 of these patients met the imaging criteria for MS. One had Dawson's fingers, which was similar to the proportion in our cohort, and 33% were initially diagnosed as MS. Of a total of 104 patients diagnosed with MS based on McDonald's DIS criteria (2010), 5 (4.8%) tested positive for MOG-Ab despite having MRI findings typical of MS and testing positive for OCB (16). It is important to be mindful that these cases were considered to be MOG-Ab positive rather than diagnosed with MS due to the good response to corticosteroid treatment and improved EDSS scores, which is more common in MOG-IgG-related disease than in MS. The poorest EDSS score was 4, which had improved to 2 on subsequent assessment (case E). However, in atypical cases with sustained disability, differential diagnoses should be considered as there is a chance of false positives for MOG-Ab.

Brain MRI findings can vary from a normal MRI to large fluffy lesions. Supratentorial lesions are more common (47%) followed by brainstem (29%) and cerebellar lesions (13%). Orbital MRI findings include unilateral or bilateral optic nerve lesions, which can be longitudinal and involve the optic chiasma. Contrast enhancement is a commonly reported feature in the optic nerve, reported in 80–100% of the cases, in association with optic nerve swelling and perineural enhancement (21–23). The spinal MRI findings in our case series are inconsistent with those of previously reported cases. Longitudinally extensive lesions with a median length of four segments are commonly reported findings in MOG-IgG-related disease (78%) (24). Hyperintense lesions on T2 involve the central gray matter of the spinal cord, producing H-shaped hyperintensity on axial MRI (9, 24–26), which was noted in case I.

Although it may be difficult to distinguish MOG-Ab positive cases from NMO seropositive cases radiologically, it has been proposed that MOG-Ab positive cases can be distinguished radiologically from MS, with high sensitivity and specificity (20). Lesions in the periventricular area, Dawson's fingers, juxtacortical U fibers, and T1 hypointense lesions are typical features of MS. In contrast, large fluffy lesions, few lesions (<3), and lesions around the third ventricle and cerebellar peduncles are more common in MOG and NMO seropositive cases (20, 27); none of our patients exhibited findings similar to these.

The optimal treatment for MOG-IgG-related disease remains controversial. Due to the lack of randomized clinical trials and rarity of the disease, the choice of immunosuppressive therapy depends on the clinical experience of the treating physician. The treatment typically follows the same approach as that for NMOSD. Azathioprine, mycophenolate, rituximab, and a longer corticosteroid taper (6 months) are all possible treatment options (11, 21).

In our case series, the patients did not receive prolonged steroid tapers. Long-term treatment was a shared patient decision; most were started on steroid sparing agents such azathioprine and mycophenolic acid, although two patients preferred not to be on any treatment.

Traditionally, it was thought that MOG-Ab-positive cases carry a benign course and good prognosis. With the expanding clinical phenotype and accumulating experience, we have learned that disability can occur, and a relapsing course is not uncommon. Patients can have severe disability at presentation, but recovery from the acute relapse may be better than that of NMO seropositive cases. Further, significant residual disability does occur (25, 28). To avoid the risk of relapses or disability, we recommended long-term immunosuppressive treatment. However, the optimal treatment regimen and duration remain unclear at this time.

Our case series provides practical and clinical indications of MOG-Ab-positive cases, which expand our knowledge of this disease. However, there are potential limitations regarding possible false-positive serology results in those with atypical presentation. The short-term follow-up of some cases and variable treatment modalities could be another limitation in this cases series.

## Conclusion

Our study demonstrates that MOG-Ab-positive cases can have a variable clinical presentation and overlap with seropositive NMO or MS cases. Relapsing courses were more frequent in our case series, and persisting disability was also observed. Distinction of this disease from others such as MS based on clinical and radiological features can be challenging. Anti-MOG testing in all patients with suggestive demyelinating events is recommended, although the possibility of false-positive tests should not be ignored. Randomized controlled trials are needed to determine the optimal treatment option and duration.

## Data Availability Statement

The datasets generated for this study are available on request to the corresponding author.

## Ethics Statement

Written informed consent was obtained from the individual(s) for the publication of any potentially identifiable images or data included in this article.

## Author Contributions

All authors listed have made a substantial, direct and intellectual contribution to the work, and approved it for publication.

### Conflict of Interest

The authors declare that the research was conducted in the absence of any commercial or financial relationships that could be construed as a potential conflict of interest.
